# Contactless Fluid Manipulation in Air: Droplet Coalescence and Active Mixing by Acoustic Levitation

**DOI:** 10.1038/s41598-018-28451-5

**Published:** 2018-07-05

**Authors:** Ayumu Watanabe, Koji Hasegawa, Yutaka Abe

**Affiliations:** 1 Graduate School of Systems and Information Engineering, University of Tsukuba, Tsukuba, Japan; 2 Department of Mechanical Engineering, Kogakuin University, Tokyo, Japan; 3 Faculty of Engineering, Information and Systems, University of Tsukuba, Tsukuba, Japan

## Abstract

Acoustic manipulation by an ultrasonic phased array provides an entirely new approach to processes such as coalescence, mixing, separation, and evaporation occurring in the generation of new materials, physical property measurement, the biomedical industry, etc. However, to date, ultrasonic phased arrays have not been fully investigated for applications in fluid manipulation. This paper provides contactless coalescence and mixing techniques for droplets in air by controlling the acoustic potential by using an ultrasonic phased array. We focused on mode oscillation to propose an efficient mixing technique for liquid without contact. A comparison of mixing performance between cases with mode oscillation and without mode oscillation showed that the flow induced by mode oscillation promotes droplet mixing. Our paper demonstrates the feasibility of contactless coalescence and mixing as a first step in fluid manipulation with a phased array.

## Introduction

Levitation technologies have recently been examined for utilization in container-less processing^1–4^. Because of its usefulness as a tool for contactless manipulation of fluids, acoustic levitation has been explored in the fields of materials science, analytical chemistry, and biomedicine^5–10^. Several approaches have been investigated for the contactless handling of matter in air. Foresti *et al*.^11^ enabled the transport and coalescence of acoustically levitated droplets by controlling the vibration velocity amplitude of each emitting element arrayed in a row. Ochiai *et al*.^12^ developed an acoustic manipulation device by using focused ultrasound transmitted from an ultrasonic phased array and enabled three-dimensional transport of solid particles by controlling the phases of transducers. Marzo *et al*.^13^ proposed a technique for freely forming arbitrary sound fields by incorporating the appropriate phase differences into an ultrasonic phased array. Nonlinear and dynamic behavior (e.g., internal and external flow of levitated droplets^14–18^, interfacial deformation and atomization^19–22^) arise in droplet manipulation by acoustic levitation. Acoustic streaming^23^ is known to be caused by a result of some acoustic energy being converted to a driving force for moving fluid. In a strong sound field in which nonlinear phenomena are observed, a droplet transforms from a sphere into an ellipsoid and is atomized^24^.

Although an ultrasonic phased array enables high precision and free generation of sound fields, most studies use solid particles, which are easy to handle, and an ultrasonic phased array is not applied for droplet manipulation. Our objective was to provide an entirely new approach for contactless fluid manipulation in air to support processes such as coalescence, mixing, separation, and evaporation. In this paper, we present a fundamental technology for levitation, coalescence and active mixing of droplets by using an ultrasonic phased array.

## Results

### Acoustic levitation by ultrasonic phased array

The acoustic manipulator is based on an ultrasonic phased array for high precision and free generation of a sound field. This device is composed of compact transducers arranged in a rectangular shape. By controlling the phase of the signal applied to each transducer, the sound waves transmitted from the transducers are focused to one point in space. The reflector faces the phased array, and the focal point of the sound generated is formed on the reflector surface. Focused ultrasound is reflected, and a localized standing wave is generated around the focal point.

**Figure 1:**
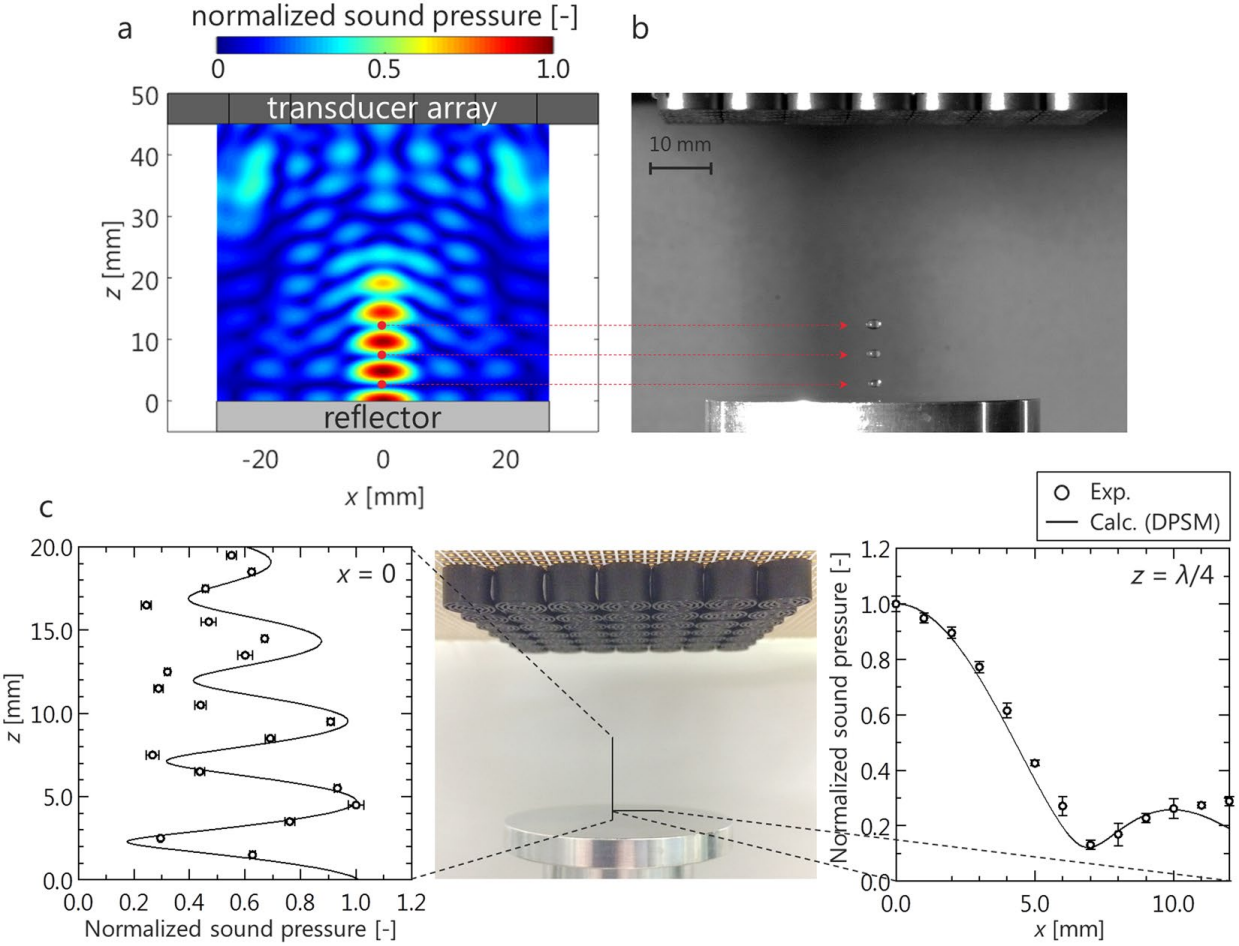
Acoustic levitation by an ultrasonic phased array. (**a**) Calculation result of acoustic field generated by focused ultrasound. (**b**) Snapshot of acoustically levitated droplets in localized standing wave. (**c**) Comparison of sound pressure between experiment and calculation (DPSM). The error bars in the experimental plot represent standard deviations.

A sound field was analyzed by the distributed point source method (DPSM)^25,26^. Details of the DPSM are described in supplementary information. Figure 1a shows the calculation results for the case in which a single focal point was formed at the intersection of the central axis of the phased array and the reflector surface. A high-pressure region was generated in a narrow area near the focal point, and the droplet was expected to be trapped in a limited area near the focal point. A snapshot of the droplets formed near the focal point is shown in Fig. 1b. Ultimately, levitation of the droplets in the localized standing wave was demonstrated. The sound pressure distribution measured by a microphone and that calculated by the DPSM are compared in Fig. 1c. These pressures are normalized by the maximum pressure. Here, *λ* is the sound wavelength of 8.5 mm. For the central axis (*x* = *y* = 0), the tendency of the sound pressure to decrease with distance from the focal point was demonstrated by both the experimental and calculation results. For the case of a pressure antinode (*z* = *λ*/4, *y* = 0), a distribution similar to the shape of a sinc function was indicated by both the experimental and calculation results. The distribution is theoretically derived based on the sound field generated by a rectangular phased array on the focal plane, which follows a sinc function^27^. This tendency was indicated by the experimental results and was reproduced by the calculation results. Therefore, the sound field generated by the phased array system can be reproduced by DPSM calculations.

### Contactless coalescence of acoustically levitated droplets by using an ultrasonic phased array

The contactless coalescence requires the generation of two focal points. Focal points were generated artificially by rapidly switching two focal points. A previous study reported that two particles were successfully levitated by switching two focal points at 500 Hz^28^. We therefore selected the switching frequency to be 500 Hz.

**Figure 2:**
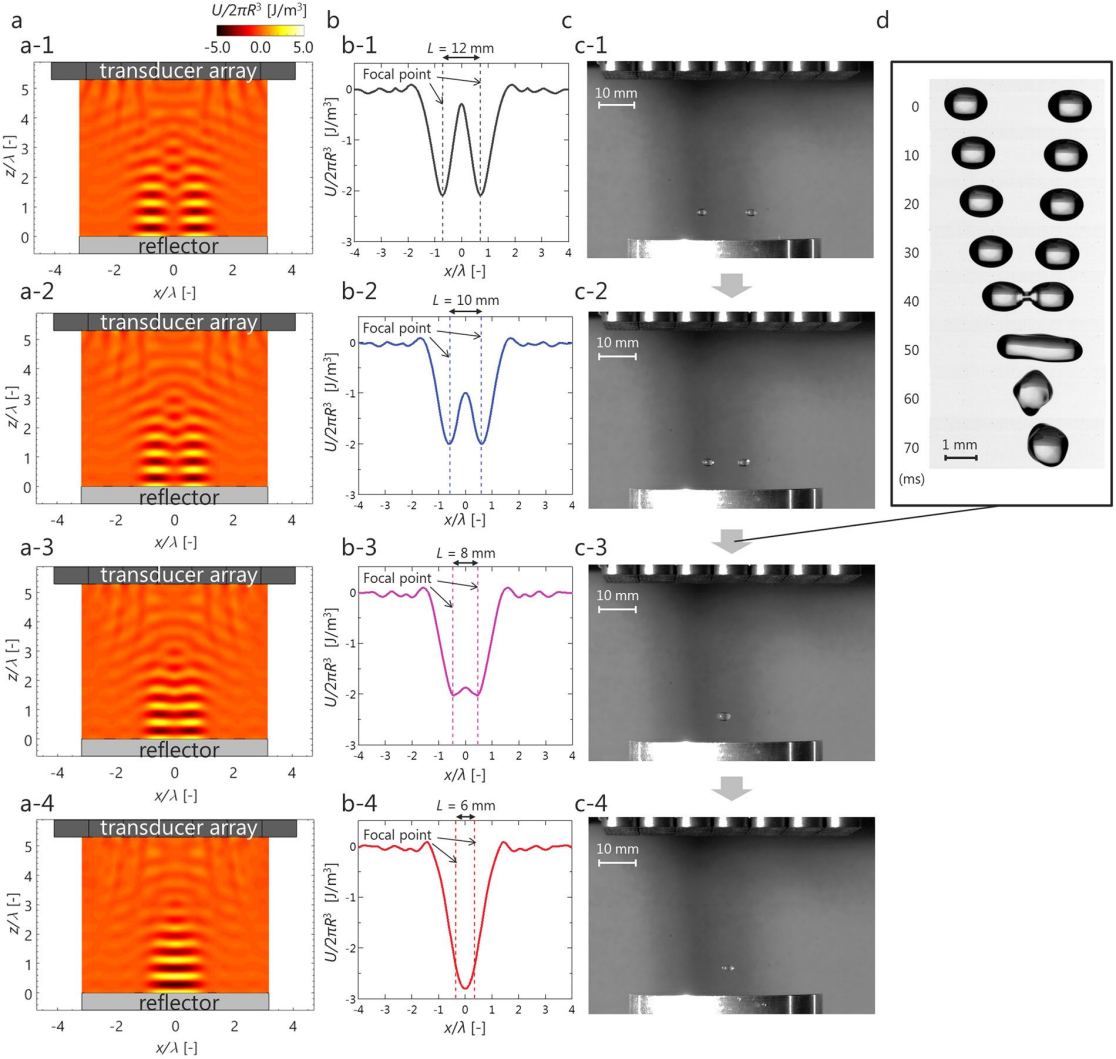
Contactless transport and coalescence by controlling the acoustic potential. (**a**) Calculation results of an acoustic potential obtained by DPSM (a-1, *L* = 12 mm; a-2, *L* = 10 mm; a-3, *L* = 8 mm; a-4, *L* = 6 mm). (**b**) Calculation results of an acoustic potential at a pressure node (*z* = 3*λ*/4) (b-1, *L* = 12 mm; b-2, *L* = 10 mm; b-3, *L* = 8 mm; b-4, *L* = 6 mm). (**c**) Snapshots of levitation behavior when a pair of focal points is generated (c-1, *L* = 12 mm; c-2, *L* = 10 mm; c-3, *L* = 8 mm; c-4, *L* = 6 mm). (**d**) Snapshot of contactless coalescence of water droplets.

**Figure 3:**
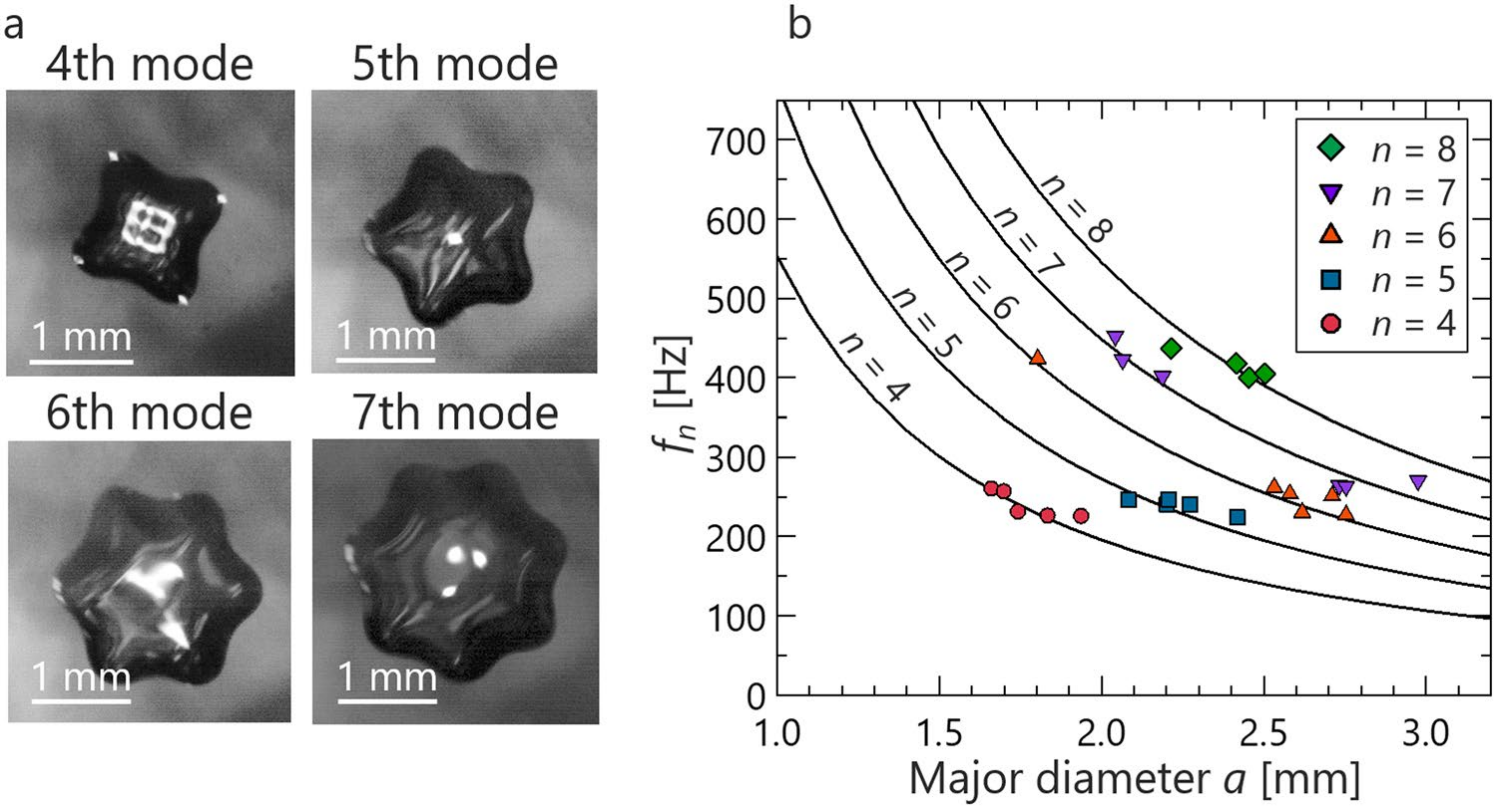
Induction of an oscillation mode by modulation of sound. (**a**) Typical 4^th^ to 7^th^ mode behavior of acoustically levitated droplet. (**b**) Condition under which oscillation mode appears. The symbols are the experimental data for the 2^nd^ to 8^th^ mode. Solid curves are the calculation results obtained using Eq. (2).

**Figure 4:**
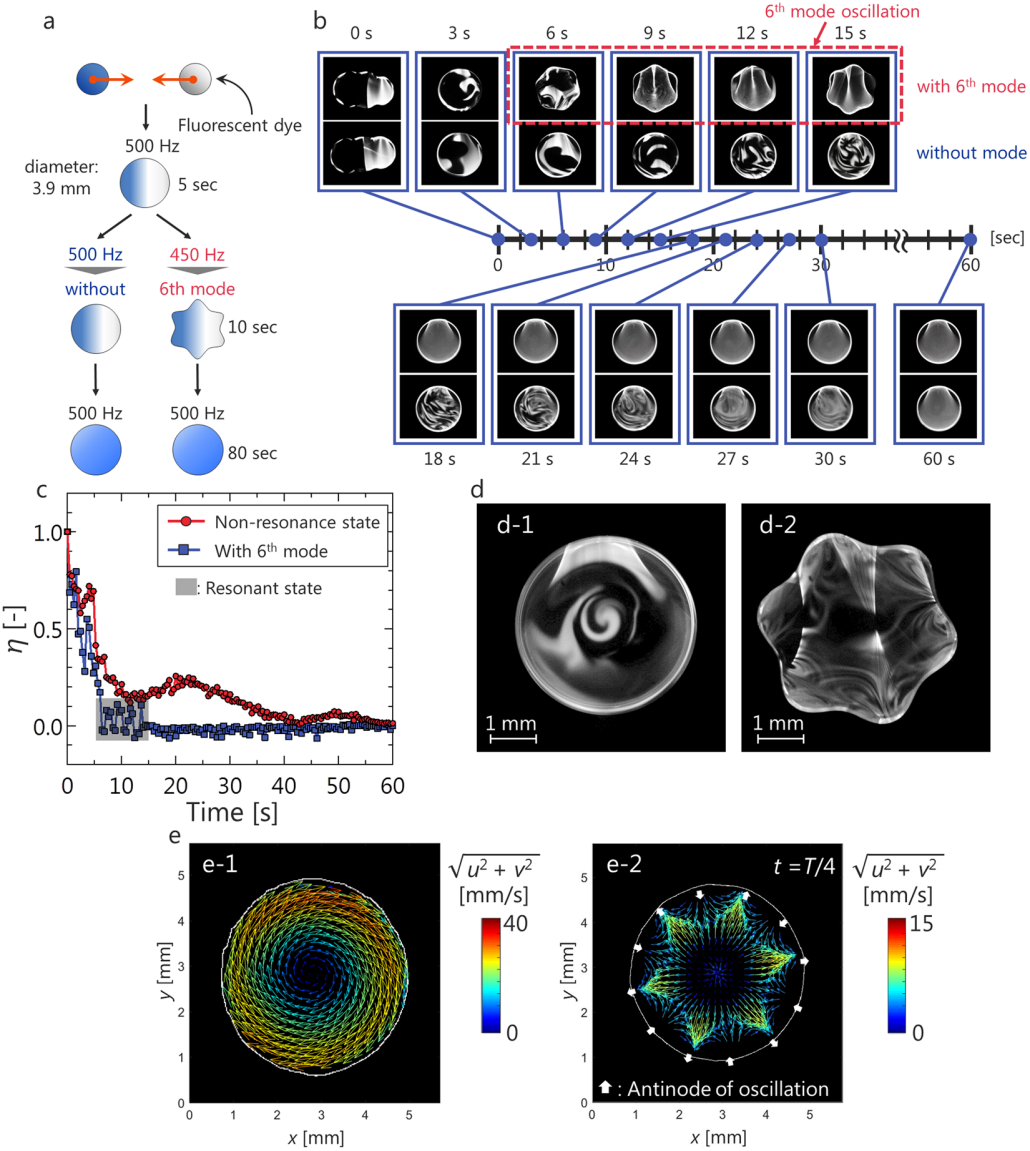
Active mixing of an acoustically levitated droplet by an oscillation mode. (**a**) Experimental procedure for observing mixing behavior. (**b**) Comparison of mixing performance between the case without a mode and the case with a mode. (**c**) Comparison of transition of mixing parameter. (**d**) Comparison of mixing pattern between (d-1) the case without an oscillation mode and (d-2) the case with an oscillation mode. (**e**) Comparison of flow structure between (e-1) the case without an oscillation mode and (e-2) the case with a 6^th^-mode oscillation.

To determine the levitation points based on the acoustic radiation force acting on levitated matter, the acoustic potential was calculated. The Gor’kov potential^29^ was defined as a field, the gradient of which gives the acoustic radiation force acting on a small sphere in a sound field:

1 $$U=2\pi {R}^{3}(\frac{{p}_{{\rm{rms}}}^{2}}{3{\rho }_{{\rm{G}}}{c}^{2}}-\frac{{\rho }_{{\rm{G}}}{v}_{{\rm{rms}}}^{2}}{2}),$$

where *R* is the radius of a small sphere, *ρ*_G_ is the density of air, *c* is the speed of sound, and *p*_rms_ and *v*_rms_ are the root mean square value of the sound pressure and particle velocity, respectively. To determine the acoustic potential, the sound pressure and particle velocity were calculated using the DPSM. Figure 2a shows the calculation results for an acoustic potential. When the spacing between the left and right focal points *L* was 12 mm (Fig. 2a-1), localized standing waves were generated at each focal point. As the distance between the focal points was reduced (Fig. 2a-2–a-4), the two standing waves formed one large standing wave. For a more detailed study, the acoustic potential at the levitation height (*z* = 3*λ*/4) was calculated, as shown in Fig. 2b. At *L* = 12 mm (Fig. 2b–1), a distinct acoustic potential well was generated at each focal point. At *L* = 10 mm (Fig. 2b–2), although the potential distribution shifted to that of a single well, two potential wells still existed at each focal point. This result is consistent with the experimental result shown in Fig. 2c-1, c-2. At *L* = 8 mm (Fig. 2b-3), the potential shape transitioned from a two shape to a single large one. This result suggests that each droplet experienced a driving force toward the center. This result is consistent with the experimental result shown Fig. 2c-3,c-4. Figure 2d and Supplementary video S1 shows the behavior observed when *L* was switched from 10 mm to 8 mm. This result demonstrates the feasibility of contactless coalescence of acoustically levitated droplets using an ultrasonic phased array.

### Contactless mixing with resonant oscillation

Active mixing techniques in air are effective for biomedical applications. Our work provides a contactless mixing technique with interface oscillation. Shen *et al*.^30^ reported that the oscillation mode of acoustically levitated droplets was induced by amplitude modulation of ultrasonic waves. The same method was used in this study. To apply oscillation to droplets without contact, the voltage applied to the transducers was modulated by 0 to 1 square wave. The modulation frequency can be tuned in 1 Hz increments. The typical oscillation behavior is shown in Fig. 3a; the figure shows bottom-view images. The test fluid was 2 cSt silicone oil. Modes were determined by the number of protrusions and then classified into the 4^th^ to 7^th^ mode. The oscillation frequency of droplets coincided within ±1% to half of the frequency of the modulation. Therefore, it is considered that the oscillation mechanism was parametric resonance, as described by Shen *et al*.^30^. To control the oscillation mode, the conditions under which the mode appears were investigated and summarized in Fig. 3b by measuring the relationship between the resonant frequency and the droplet diameter. The solid curve is described by replacing the diameter of the Rayleigh equation^31^ with the major diameter:

2 $${f}_{n}=\frac{1}{2\pi }\sqrt{\frac{8{\sigma }_{{\rm{L}}}}{{\rho }_{{\rm{L}}}{a}^{3}}n(n-1)(n+2)}$$

where *σ*_L_ is surface tension, *ρ*_L_ is the density of a droplet, *a* is the major diameter, and *n* is the oscillation mode. The theoretical value yielded by eq. (2) is reflected in the experimental results. Therefore, the Rayleigh equation, assuming a spherical droplet, can be extended to an acoustically levitated droplet by adopting the major diameter as the diameter. This finding agrees well with Shen’s result^30^ and shows that an oscillation mode is governed by the Rayleigh equation.

Differences in mixing behavior between the case with a mode and the case without a mode were investigated by laser-induced fluorescence (LIF). A 50 wt% glycerin aqueous solution was used for the observation of mixing behavior. Because the fluctuations of a viscous droplet after collision can be suppressed as much as possible, it is expected that the observation of mixing behavior is clear. The measurement procedure is shown in Fig. 4a. A droplet containing a fluorescent dye and a droplet without the dye coalesced and were levitated for 5 seconds to remove the disturbance caused by coalescence. The droplets were irradiated from one side with a Nd:YAG sheet laser, and fluorescence emission was observed via a high-speed video camera from the bottom. The major diameter after coalescence was adjusted to approximately 3.9 mm. According to the Rayleigh eq. (2), modes are not induced at an oscillation frequency of 500 Hz at this diameter, but the 6^th^ mode is induced at an oscillation frequency of 450 Hz. Therefore, we compare the cases with oscillation frequencies 450 Hz and 500 Hz after coalescence for 10 seconds.

Figure 4b shows the observation results pertaining to mixing behavior. In the case without a mode, the luminance distribution became uniform 60 seconds after coalescence (Supplementary video S2). On the other hand, in the case with the 6^th^ mode, the luminance become uniform within 10 seconds after the mode appeared (Supplementary video S3). The mixed state was evaluated by the mixing parameter *η*^32^. Based on the LIF results, the average *μ* and the standard deviation *σ* of the luminance in the *N* pixels mixed region were calculated. One standard deviation indicates the difference from the fully mixed state. The mixing parameter *η* is defined by using the normalized standard deviation:

3 $$\eta =\frac{{(\frac{\sigma }{\mu })}_{t}-{(\frac{\sigma }{\mu })}_{t=\infty }}{{(\frac{\sigma }{\mu })}_{t=0}-{(\frac{\sigma }{\mu })}_{t=\infty }}.$$

The mixing parameter begins from *η* = 1 and approaches *η* = 0 as mixing progresses. The measurement result of the time trace of *η* is shown in Fig. 4c. The image obtained when a droplet collided was used for *t* = 0, and the image obtained after 10 minutes—which confirmed that mixing was completed by observation—was used for *t* = $\infty$. In the case without an oscillation mode, *η* converged to zero after approximately 60 seconds. On the other hand, *η* converged to zero after 15 seconds, that is, within 10 seconds after the mode appeared in the case with the 6^th^ mode. These results show that mixing of droplets can be promoted without contact by using an oscillation mode.

A characteristic mixing pattern is shown in Fig. 4d. In the case without a mode (Fig. 4d-1), the mixing pattern showed a swirling characteristic at the center of the droplet. In the case with an oscillation mode (Fig. 4d-2), a vortex-like pattern was formed at the antinode of the oscillation. To investigate why the mixing behavior varied, the internal flow structures of droplets were compared by particle image velocimetry (PIV). A 50 wt% glycerin aqueous solution was used as the test fluid. The droplets were irradiated from one side by a Nd:YAG sheet laser, and the fluorescence emission of the particles was observed via a high-speed video camera from the bottom. Figure 4e-1 shows the PIV result for the case without oscillation. Rotational flow around one axis occurred inside the droplet. This is the same flow structure found in acoustically levitated glycerol droplets^15^. Figure 4e-2 shows the PIV result for the case with the 6^th^ mode. To clearly observe the flow induced by interface oscillation, the rotational component of the droplet was removed based on the rotational speed measured by tracking the antinode of the oscillation. The white arrow in Fig. 4e B indicates the position of the oscillation antinode and the movement direction of the interface. Near the oscillation antinode, flow occurred toward the same direction of the interface displacement. It was clarified that flow different from that in the non-oscillation state was induced by interface oscillation.

## Discussion

A pair of droplets was successfully levitated by generating two acoustic potential wells by controlling the phase of sound. Based on potential estimation (Fig. 2b), it was clarified that the potential shape changed from a two shape to a single large shape by decreasing the gap between the two focal points, and the levitated droplets then moved toward the center and eventually coalesced. It is considered that the conditions under which the droplets coalesce can be predicted by the Gor’kov potential. In this report, although the phase difference at the arbitrary point where sound is focused (the value was calculated based on the geometrical arrangement and speed of sound) was used, by applying the optimal acoustic trap proposed by Marzo *et al*.^13^, further innovations such as three-dimensional manipulation, giving droplets an angular momentum, and adjustment of the retention force and driving force are expected.

After coalescence, the droplets could be mixed by the oscillation mode. Based on the visualization results pertaining to mixing behavior (Fig. 4), the fluorescence dye inside the droplet was homogenized within 10 seconds after the oscillation mode appeared and in approximately 60 seconds without an oscillation mode. Here, we compared the order of diffusion mixing and convection mixing. The diffusion coefficient^33^ was roughly *D* ∼ 10^−4^ mm^2^/s, and the characteristic distance between the droplets was *l* ∼ 10^0^ mm; thus, the characteristic time of diffusion was *t*_θ_ ∼ *l*^2^/*D* ∼ 10^4^ seconds. Consequently, regardless of the oscillation mode, convection is more dominant than diffusion. For the case without an oscillation mode, it is considered that the inertia of coalescence and acoustic streaming inside the droplets promoted mixing. On the other hand, for the case with an oscillation mode, mixing behavior changed drastically by inducing the oscillation mode. Observation results showed that when an oscillation mode appeared, near the antinode of an oscillating droplet, stretching and folding of the interface by the action of a flow^34^ was observed, and it progressed toward the inside of the droplet. It is considered that the formation of vortices via stretching and folding induced by oscillation promotes mixing.

These results offer the potential of creating a platform for contactless fluid manipulation technologies and fundamental fluid science, including not only applied approaches to engineering but also database creation with respect to droplet dynamics. Future studies should address the control over the size of injected droplets and the handling of microscale droplets for biomedical, lab-on-a-drop, and other applications. These fields also require expansion of the technology toward handling processes, including separation and phase change.

## Methods

### Experimental setup

Acoustic levitation is achieved by a standing wave at the focal point of sound. By transmitting sound waves with a controlled phase, the focal point of sound is generated at an arbitrary position. By reflecting the focused ultrasound using a reflector, a localized standing wave can be generated near the focal point.

**Figure 5:**
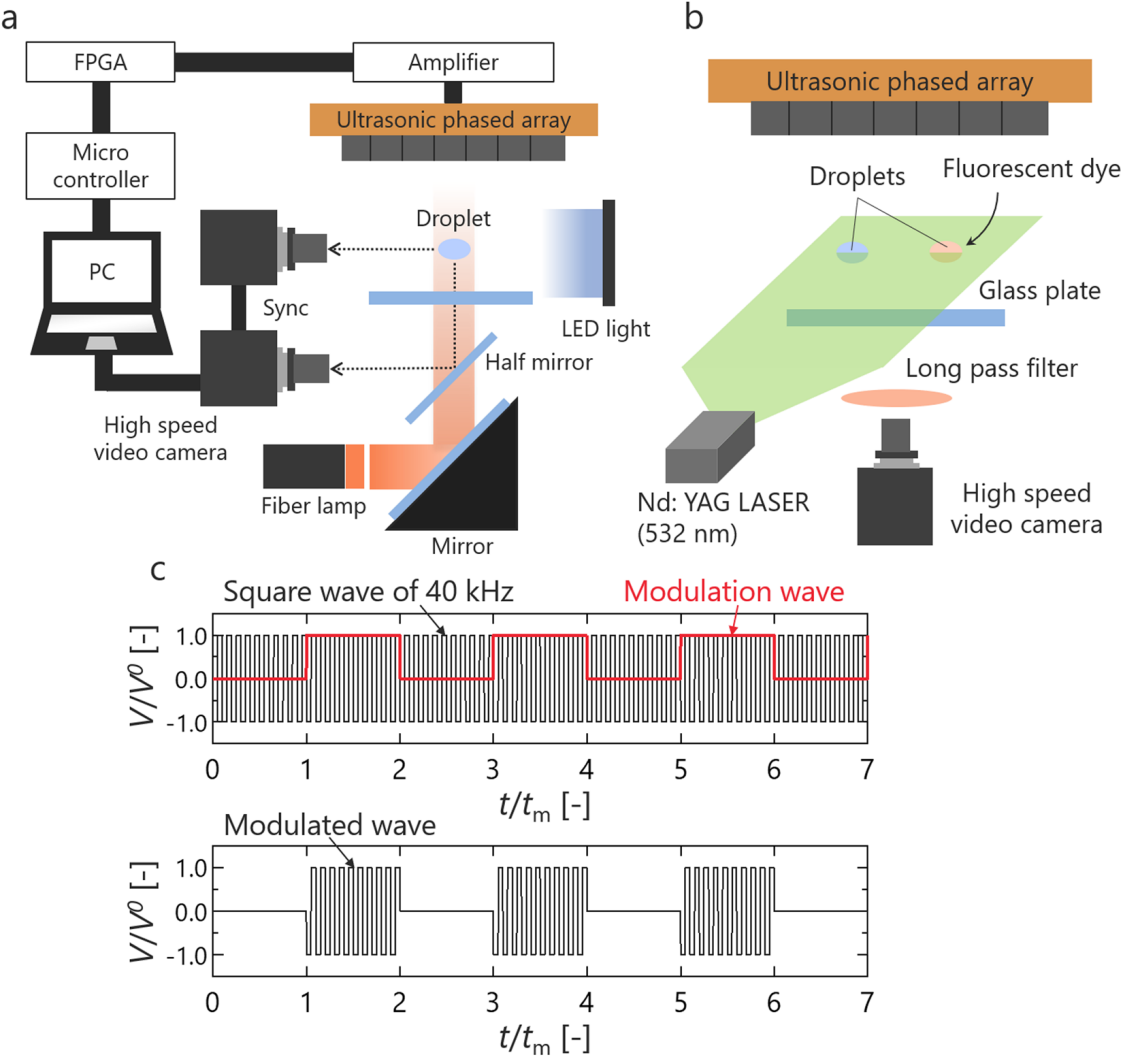
Schematic diagram of experimental apparatus. (**a**) The experimental system for observing droplet behavior. (**b**) The experimental PIV and LIF system. (**c**) Modulation of the voltage applied to the transducers. *V* is the voltage, *V*o is the voltage amplitude, and *T*_m_ is the modulation period.

We used a 7 × 7 square transducer array consisting of 49 small ultrasonic transducers. The diameter of transducer was 10 mm, the frequency was 40 kHz. Phase control of the sound transmitted from each transducer is required to generate an ultrasonic focal point. We realized this control using a field programmable gate array (FPGA) (Altera Co., Cyclone-IV DE0-Nano). The experimental apparatus is shown in Fig. 5. Both the focal length and distance from the transducer to the reflector were 45 mm. The ambient temperature and relative humidity were kept at 20 ± 3 °C and 40 ± 3%, respectively. Typical sound pressure levels were tuned between 155 and 159 dB for ensuring stable levitation of the droplet. The sound pressure was measured a probe microphone (Bryel & Kjaer, Type 4138, Diameter: 1/8 inch) for the quantitative evaluation of the sound field. The microphone was fixed on the traverse device, which could move along the *x*, *y*, and *z* directions. The behavior of levitated droplets was observed by back-light illumination from the side and coaxial declination from the bottom via a high-speed video camera (Photron Co., Ltd. FASTCAM-Mini UX100) (Fig. 5a). The state inside the droplet was observed with a Nd:YAG sheet laser with a wavelength of 532 nm (Japan Laser Co., DPGL-5W-L) irradiated from the side of the droplets (Fig. 5b). Fluorescent particles (EBM Co., FLUOSTAR®) were used for the observation of internal flow, and a fluorescent dye (Kanto Kagaku Co., Rhodamine 6G) was used to observe the mixing behavior. To remove scattered light, a long-pass filter (Kenko Tokina Co., YA3) was used. Figure 5c shows the signal waveform applied to the transducers when applying oscillation to droplets. The voltage applied to the transducers was modulated from 0 to 1 square wave. Table 1 lists the test fluids and their physical properties. Three fluids of varying density, surface tension and viscosity were selected.

**Table 1:** Properties of test fluids^35^.

Sample	*ρ*_L_ (kg/m^3^)	*μ*_L_ (10^−3^ Pa · s)	*σ*_*L*_ (mN/m)
Water	998	1.00	72.7
2 cSt Silicone oil (Shin-Etsu Chemical Co., KF-96)	873	1.75	18.3
50 wt% Glycerin aqueous solution	1125	6.03	70.0

### Statistical analysis

The error of the droplet diameter could be a maximum of <5% because the levitated droplets were observed with a spatial resolution of 10 ± 5 μm/pix. Sound pressure was measured 3 times, and the error was a maximum of <7%.

## Electronic supplementary material

Supplementary information

S1. Contactless coalescence of acoustically levitated water droplets by an ultrasonic phased array

S2. Mixing behavior inside the droplet without mode

S3. Mixing behavior inside the droplet with 6th mode
