# Supplementary material for: Contactless Fluid Manipulation in Air: Droplet Coalescence and Active Mixing by Acoustic Levitation
Source: Sci Rep. 2018 Jul 5;8:10221. doi: 10.1038/s41598-018-28451-5 (PMC6033947; doi:10.1038/s41598-018-28451-5)
Supplement: Supplementary file 1 — Supplementary information [file 41598_2018_28451_MOESM1_ESM.pdf]

Supplementary information
Contactless Fluid Manipulation in Air:
Droplet Coalescence and Active Mixing by Acoustic Levitation

Ayumu Watanabe[1], Koji Hasegawa[2], and Yutaka Abe[3].

[1]Graduate School of Systems and Information Engineering, University of Tsukuba, Tsukuba, Japan.

[2]Department of Mechanical Engineering, Kogakuin University, Tokyo, Japan.

[3]Faculty of Engineering, Information and Systems, University of Tsukuba, Tsukuba, Japan.

Calculation method of sound field

The distributed point source method (DPSM)[1, 2] is used to model a sound field without a computational mesh by distributing point sources of sound at the boundary. Emitters and reflectors are discretized by point sources to represent the sound field. The sound pressure p_{m} and the velocity v_{m} at a distance r_{mn} generated by a sound wave radiated from the point source m is given by

$$p_{\mathrm{m}}(r_{\mathrm{mn}}) = A_{\mathrm{m}} \frac{\exp i(kr_{\mathrm{mn}} - \omega t_{\mathrm{m}})}{r_{\mathrm{mn}}} = A_{\mathrm{m}} G(r_{\mathrm{mn}}), \tag{S1}$$

$$v_{\mathrm{m}}(r_{\mathrm{mn}}) = \frac{\boldsymbol{n} \cdot \boldsymbol{r}_{\mathrm{mn}}}{i\omega\rho_{\mathrm{G}}} \frac{\partial p}{\partial r} = A_{\mathrm{m}} M(r_{\mathrm{mn}}), \tag{S2}$$

where A_{m} is the strength of the m-th point, $k = 2\pi/\lambda$ is the wavenumber, ω is the angular frequency and ρ_{G} is the density of air. For an ultrasonic phased array, sound waves radiated from each transducer are excited at different time intervals Δt_{m} to focus sound waves. Thus, the term t_{m} in Eq. (S1) can be expressed in terms of the phase difference from a reference time t:

$$t_{\mathrm{m}} = t - \Delta t_{\mathrm{m}}. \tag{S3}$$

If there are N point sources, the total value at point x is given by

$$p(x) = \sum_{\mathrm{m}=1}^{\mathrm{N}} p_{\mathrm{m}}(r_{\mathrm{mn}}), \quad \text{(S4)}$$

$$v(x) = \sum_{\mathrm{m}=1}^{\mathrm{N}} v_{\mathrm{m}}(r_{\mathrm{mn}}). \quad \text{(S5)}$$

If there is a secondary sound source such as a reflector, the amplitudes of the sound sources are determined to satisfy the boundary condition. N point sources whose amplitude is unknown are distributed over both the primary sound source (e.g., transducers) and the secondary sound source (e.g., reflector). Here, it is assumed that the sound sources are spheres of radius r_{s}, and the radiation points are located at positions retracted by r_{s} from the boundary. By giving the velocity V_0 to the primary sound sources and velocity 0 to the secondary sources, the following equation is obtained:

$$\{v_{\mathrm{m}}\} = {}^{t}\{V_0, \cdots V_0, 0, \cdots 0\}, \quad \text{(S6)}$$

$$\{A_{\mathrm{m}}\} = [M_{\mathrm{mn}}]^{-1}\{v_{\mathrm{m}}\}. \quad \text{(S7)}$$

Because N equations are given for N unknown sound sources, the amplitudes of the unknown sound sources are determined.

Supplementary Video S1

This video shows the contactless coalescence of acoustically levitated droplets by an ultrasonic phased array. The record speed was 8000 fps, and the play speed was 60 fps.

Supplementary Video S2

This video shows the mixing behavior inside the droplet without mode. The record speed was 500 fps, and the play speed was 240 fps.

Supplementary Video S3

This video shows the mixing behavior inside the droplet with 6th mode. The record speed was 500 fps, and the play speed was 240 fps.

References

1. Ahmad, R., Kundu, T. and Placko, D., Modeling of phased array transducers, *J. Acoust. Soc. Am*., **117**, 1762-1776 (2005).

2. Wada, Y., Yuge, K., Nakamura, R., Tanaka, H. & Nakamura, K., Dynamic analysis of ultrasonically levitated droplet with moving particle semi-implicit and distributed point source method, *Jpn. J. Appl. Phys*., **54**, 07HE04 (2015).
